# Cigarette smoke extract stimulates bronchial epithelial cells to undergo a SUMOylation turnover

**DOI:** 10.1186/s12890-020-01300-w

**Published:** 2020-10-23

**Authors:** Haifeng Zhou, Lei Zhang, Yang Li, Guorao Wu, He Zhu, Huilan Zhang, Jia-Kun Su, Lei Guo, Qing Zhou, Fei Xiong, Qilin Yu, Ping Yang, Shu Zhang, Jibao Cai, Cong-Yi Wang

**Affiliations:** 1 The Center for Biomedical Research, Tongji Hospital Research Building, Tongji Hospital, Tongji Medical College, Huazhong University of Science and Technology, Wuhan, Caidian, 431000 China; 2 Department of Respiratory and Critical Care Medicine, Tongji Hospital, Tongji Medical College, Huazhong University of Science and Technology, Wuhan, China; 3 The Technology Center, China Tobacco Jiangxi Industrial Co., Ltd., Nanchang High Technology Development Valley, Nanchang, 330096 China

**Keywords:** Chronic obstructive pulmonary disease, Posttranslational modification, SUMOylation, Cigarette smoke extract, Oxidative stress

## Abstract

**Background:**

Chronic obstructive pulmonary disease (COPD) characterized by the airway and lung inflammation, is a leading cause of morbidity and mortality worldwide, especially among smokers over 40 years of age and individuals exposed to biomass smoke. Although the detailed mechanisms of this disease remain elusive, there is feasible evidence that protein posttranslational modifications (PTMs) may play a role in its pathoetiology. We thus conducted studies to dissect the effect of cigarette smoke extracts (CSE) on the change of SUMOylated substrates in human bronchial epithelial cells (HBEs).

**Methods:**

Samples were collected in HBEs with or without 24 h of CSE insult and then subjected to Western-blot and LC-MS/MS analysis. Subsequently, bioinformatic tools were used to analyze the data. The effect of SUMOylation on cytochrome P450 1A1 (CYP1A1) was evaluated by flow cytometry.

**Results:**

It was noted that CSE stimulated HBEs to undergo a SUMOylation turnover as evidenced by the changes of SUMOylated substrates and SUMOylation levels for a particular substrate. The SUMOylated proteins are relevant to the regulation of biological processes, molecular function and cellular components. Particularly, CSE stimulated a significant increase of SUMOylated CYP1A1, a critical enzyme involved in the induction of oxidative stress.

**Conclusions:**

Our data provide a protein SUMOylation profile for better understanding of the mechanisms underlying COPD and support that smoking induces oxidative stress in HBEs, which may predispose to the development of COPD in clinical settings.

## Background

Chronic obstructive pulmonary disease (COPD), which affects more than 10% of population over 40 years of age, is a leading cause of hospital admissions and is currently the third leading cause of death worldwide [1]. The basic characteristics of COPD are chronic bronchiolitis and emphysema. Patients who have chronic bronchiolitis show persistent inflammation, goblet cell hyperplasia, and mucin hyperexpression in the airway [2]. The process of inflammation in COPD is characterized by the increased infiltrated T cells, B cells, neutrophils, dendritic cells and macrophages along with the upregulation of proinflammatory cytokines, leading to the destruction of lung tissue coupled with a decline in pulmonary function [3–5]. Accumulated evidence shows that cigarette smoking is a key risk factor for COPD, and cigarette smoke extract (CSE), which contains more than 4000 different types of constituents, including many toxic oxidants, is capable of inducing oxidative stress in airway epithelial cells, and ultimately, damaging airway epithelial cells [6, 7].

Posttranslational modifications (PTMs) are a common and reversible regulatory modification that allows the cells being able to regulate the function of proteins in response to intra- and extracellular signals. SUMOylation, one of the evolutionarily conserved reversible PTMs, is featured by the covalent attachment of the small ubiquitin-like modifier (SUMO) to its substrate proteins. In general, SUMO conjugates to its substrates involving an activating (E1) enzyme (a heterodimer known as SAE1/SAE2), a conjugating (E2) enzyme (Ubc9) that form thioesters (S) with the modifiers, and an E3 ligase [8, 9]. Moreover, as a reversible modification, SUMO can be removed from the target proteins by SENPs (SENP1–7), which called de-SUMOylation. SUMOylation is employed by almost all eukaryotes to regulate many cellular processes such as gene expression, genome stability, DNA damage repair, RNA processing, cell cycle progression and quality control of newly synthesized proteins [8, 10].

A growing number of studies have shown that the imbalance of SUMOylation and deSUMOylation is associated with the occurrence and progression of various diseases such as cancer, degenerative diseases, and diabetes [11–13]. Particularly, we have recently shown that SUMOylation is required to protect pancreatic beta cells against oxidative stress, partly by regulating the activity of nuclear factor-erythroid 2-related factor 2 (NRF2) to enhance reactive oxygen species (ROS) detoxification [14]. Given the role of NRF2 in the pathogenesis of COPD, we thus hypothesize that SUMOylation is of great importance in the pathogenesis of COPD [15]. To address this issue, we created a profile of differentially SUMOylated proteins by LC-MS/MS analysis in human bronchial epithelial cells (HBEs) following CSE challenge. We characterized that CSE stimulated HBEs to undergo a SUMOylation turnover characterized by the changes of SUMOylation patterns of substrates or SUMOylation levels for a particular substrate, which are implicated in the regulation of biological processes, molecular function and cellular components. In particular, our study revealed that CSE significantly enhanced CYP1A1 SUMOylation, indicating that oxidative stress induced by smoking plays a critical role in HBE injury, which may contribute to the initiation and progression of COPD.

## Methods

### Cell culture and treatment

The human Bronchial epithelial cell (HBE) line was obtained from the Shanghai Institute of Cell Biology, Chinese Academy of Sciences (Shanghai, China) and maintained in Dulbecco’s Modified Eagle’s Medium (DMEM, Gibco, Shanghai, China) with 10% fetal bovine serum (FBS, Hyclone, Rockford, IL, USA), 50 μg/mL penicillin, and 50 μg/mL streptomycin (Sigma, St Louis, MO, USA). The cultures were maintained in a humidified atmosphere of 5% CO2 at 37 °C. Once cells reached 70–80% confluence, 20% CSE was added into the culture medium, and an equal volume of PBS was applied as a control for 24 h.

### Preparation of CSE

CSE was prepared as described previously [16]. Briefly, mainstream smoke from one cigarette (Jiangxi Industrial Co., Ltd., Nanchang, China) was bubbled through 10 ml of culture medium. After adjustment of the pH to 7.4, the CSE was sterile-filtered through a 0.22-μm filter (33-mm Millex GV; Merck Millipore, Billerica, MA). This solution was considered to be 100% CSE. CSE was standardized by measuring the absorbance at wavelength 320 nm, freshly prepared for each experiment, and diluted with culture medium supplemented with 10% FBS before use.

### Co-immunoprecipitation

Co-immunoprecipitation was carried out to confirm that CSE challenged HBEs underwent a change in terms of total SUMOylation levels or patterns. HBEs with or without CSE treatment were lysed in 1X RIPA cell lysis buffer containing a protease inhibitor cocktail (Sigma, St Louis, MO, USA) and phosphatase inhibitors A and B (Sigma, St Louis, MO, USA) in the presence of 20 mmol/L N-ethylmaleimide (NEM, Sigma, St Louis, MO, USA). The protein concentration was measured using the Bradford protein assay according to the manufacturer’s instructions (Boster Biological Technology co. ltd, Pleasanton, CA, USA). Endogenous SUMO1 and their associated substrates were then immunoprecipitated using a SUMO1 antibody (Cell Signaling, Danvers, MA) as reported [14], and the resulting precipitants were subjected to quantitative Western blot analysis as described below.

### Sample collection and LC-MS/MS

Two independent samples of HBEs were harvested following 24 h of CSE insult and then subjected to LC-MS/MS analysis (PTM-Biolabs Co. Ltd., Hangzhou, China). The corresponding cells treated with vehicle were served as controls. Briefly, the SUMO-1 antibody affinity enrichment columns (Acclaim PepMap 100, Thermo Scientific) were employed to pull-down the SUMOylated proteins, followed by a label-free proteomic analysis to quantify SUMO1-cojugated lysine residues. Each sample was undergone three parallel LC-MS/MS analyses to obtain accurate quantification results.

### Bioinformatics analysis

Gene Ontology (GO) annotation proteome derived from the UniProt-GOA database (http://www.ebi.ac.uk/GOA/) was employed for proteomic data analysis. Briefly, the identified protein ID was converted into UniProt ID and then mapped to GO IDs. If some of the identified proteins were not annotated by the UniProt-GOA database, the InterProScan software would be used to annotate protein’s GO properties based on protein sequence alignment method. The proteins were then classified by Gene Ontology annotation based on three categories: biological process, cellular component and molecular function. Kyoto Encyclopedia of Genes and Genomes (KEGG) database (http://www.genome.jp/kegg/) was next used to annotate protein pathway. Firstly, the KEGG online service tool, KAAS, was used to annotate the description of protein function in the KEGG database, and the annotated results were mapped to the KEGG pathway database using the KEGG online service tool, KEGG mapper. For each category, a two-tailed Fisher’s exact test was employed to test the enrichment of the differentially pulled down proteins against all identified proteins. Corrected *P* value< 0.05 was considered significant when performing the bioinformatic analysis. The wolfpsort software was used to predict subcellular localization for a particular identified protein.

### Western blot analysis

The above prepared cell lysates or immunoprecipitates were separated on 10% (vol/vol) polyacrylamide gels and transferred onto PVDF membranes. The lysates and imminoprecipitates were probed with primary antibodies, including SUMO1, SUMO2/3 (Cell Signaling, Danvers, MA, USA), SUMO-specific protease 3 (SENP3), SUMO-specific protease 7 (SENP7) and beta-actin (Santa Cruz, Delaware, California, USA), and UBC9 (Cell Signaling, Danvers, MA, USA) for quantitative Western blot analysis, respectively, to confirm that the CSE insulted HBEs underwent a change in terms of total SUMOylation levels or patterns [14]. Similarly, to verify that CYP1A1 was SUMOylated by SUMO1, the above precipitates were probed with a CYP1A1 antibody (13241–1-AP) (Proteintech Group, Inc., Wuhan, China) and the reactive bands were developed using the established techniques [17]. Briefly, the membranes were incubated with an indicated primary antibody at 4 °C overnight. After washes with TBST (0.5% Tween) five times, the membranes were probed with an appropriate HRP-conjugated secondary antibody for 1 h, and the reactive bands were visualized using the ECL reagents (Servicebio, Wuhan, China) as instructed. Quantitative analysis of relative intensity of each band was conducted using the Image J software and β-actin was used for normalization.

### CYP1A1 enzymatic activity assay

CYP1A1 enzymatic activity was measured in HBEs using a P450-Glo CYP1A1 Assay kit (Promega, Madison, WI, USA). All assays were performed in 96-well white-walled plates with clear bottoms (Greiner Bio-One, Kremsmünster, Austria) using the method as described by the manufacturer. The assay was performed on cells at 70–90% confluence.

### Measurement of ROS

Measurement of reactive oxygen species (ROS) was carried out using a ROS assay kit (Beyotime, Shanghai, China) as previously described [18]. The cells were seeded at a density of 2.5 × 10^5^ cells/well in 6-well culture plates in a final volume of 2 mL of DMEM with 1% FBS and were grown to ~ 70 to 80% confluency. Freshly prepared CSE was added, and the cells were incubated for 24 h, washed 3 times with prewarmed PBS, resuspended in 1 mL of PBS, and incubated with or without DCFH-DA (10 μM) for 30 min. The cells were washed and resuspended in PBS, and flow cytometric analysis was performed using a BD LSR flow cytometer (BD Biosciences, San Jose, CA, USA).

### Apoptosis analysis

HBE apoptosis was analyzed using a PE Annexin V Apoptosis Detection Kit (BD Pharmingen, NA, USA) following the manufacturer’s instructions. The antioxidant Tempol (1 mM, Sigma-Aldrich) was added to rescue the injury induced by CSE treatment. The cells were treated and processed as described above, followed by addition of 5 uL PE-Annexin V and 5 uL 7-AAD solution to each well. After incubation at room temperature in the dark for 15 min, the cells were analyzed under a BD LSR flow cytometer (BD Biosciences, San Jose, CA, USA) as instructed.

### Statistical analysis

All the experiments were conducted with at least three independent replications, except for the LC-MS/MS analysis, in which only two independent samples were used. Data are expressed as the mean ± SEM. All statistical analyses were carried out using GraphPad Prism 5.0 software (GraphPad Software Inc., San Diego, CA, USA). Data were analyzed using the Student’s *t*-test. A *P* value ≤0.05 was considered statistically significant.

## Results

### CSE induces HBEs to undergo a SUMOylation turnover

To demonstrate the effect of cigarette smoking on the changes of SUMOylated proteins in HBEs, we examined the expression of SUMO proteins and SUMOylation associated enzymes following CSE stimulation. To this end, we first sought to optimize the CSE dose and exposure time by serial of dilution assays. It was noted that CSE induced a significant increase in terms of SUMO1 expression following 20% CSE exposure for 12 h, and lasted up to 48 h **(**Supplementary Fig. 1). Furthermore, HBEs underwent apoptosis following 24 h of 20% CSE treatment (Supplementary Fig. 2), and a dose-dependent induction of SUMO1 expression was noted, but it became absent once CSE concentration reached above 20%. We thus selected 20% CSE and 24 h of exposure time for our experimental purpose.

**Fig. 1:**
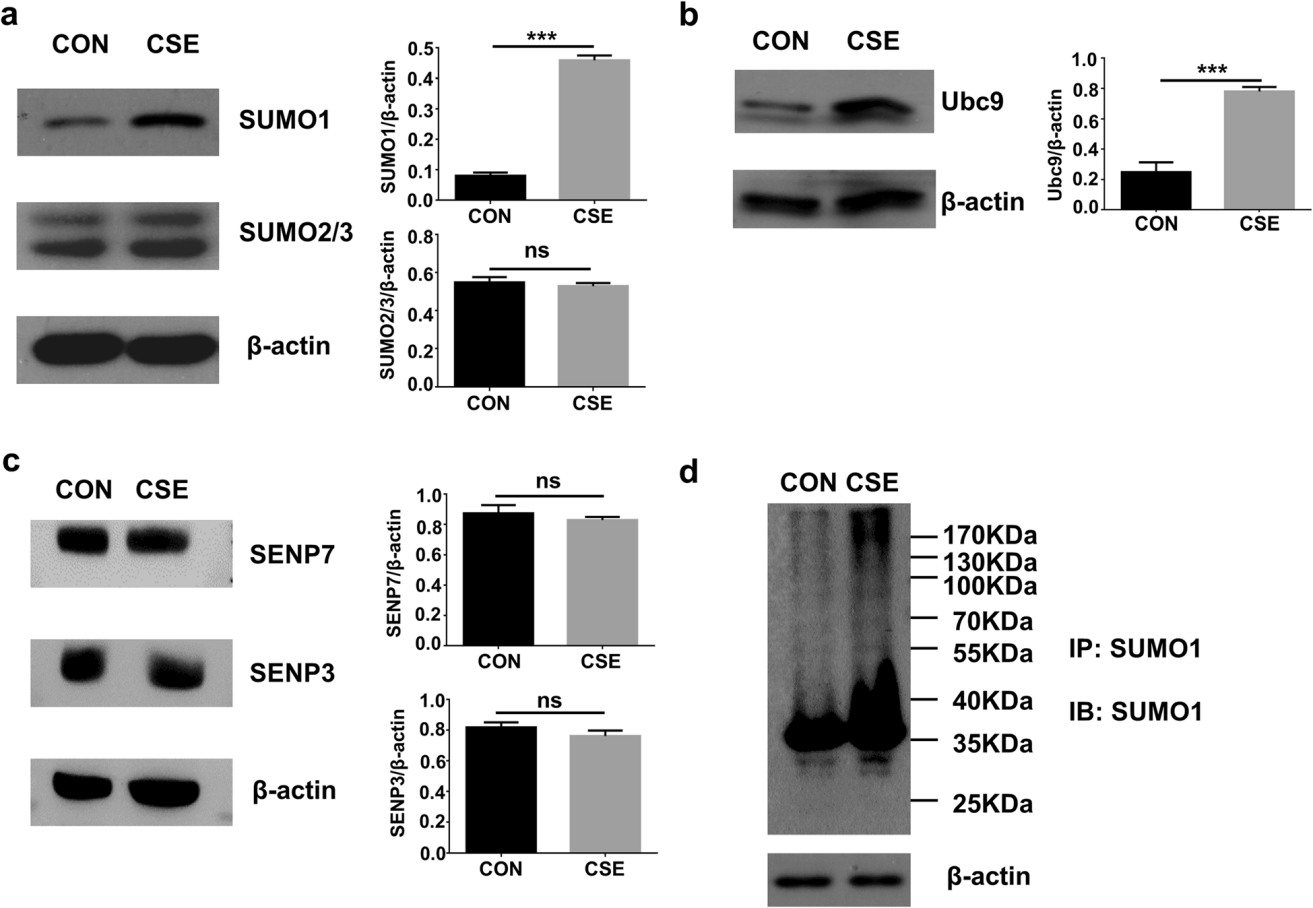
CSE selectively induces increased expression of SUMO1 and Ubc9. HBEs challenged with CSE manifested enhanced SUMO1 (**a**) and Ubc9 **(b)** expression, but without perceptible change for Senp3 and 7 **(c)**. Left panel: Representative results for Western blot analysis; r ight panel: quantitative results derived from 3 independent replications. **d** Western blot results of Co-IP products, which indicated increased SUMOylation profile by SUMO1 following CSE insult in HBEs. **, *P* < 0.01; ***, *P* < 0.001

Interestingly, Western blot analysis of HBE lysates found that CSE induced significantly higher levels of SUMO1 rather than SUMO2/3 expressions **(**Fig. 1a**)**. Similarly, CSE challenge dramatically increased the expression of Ubc9, the only conjugating enzyme required for SUMOylation in HBEs **(**Fig. 1b**)**, but no perceptible change was detected for the expression of SENP3 and SENP7, the two enzymes necessary for deSUMOylation **(**Fig. 1c**)**. The remarkable up-regulation of SUMO1 and Ubc9 prompted us to check the SUMOylation patterns by SUMO1. In consistent with our observation, Western blot analysis of immunoprecipitates pulled down by a polyclonal SUMO-1 antibody revealed that CSE insult rendered HBEs to undergo the changes of SUMOylation patterns of its substrates and SUMOylation levels for a particular substrate **(**Fig. 1d**)**.

### Characterization of SUMOylated substrates in HBEs by LC- MS/MS

**Fig. 2:**
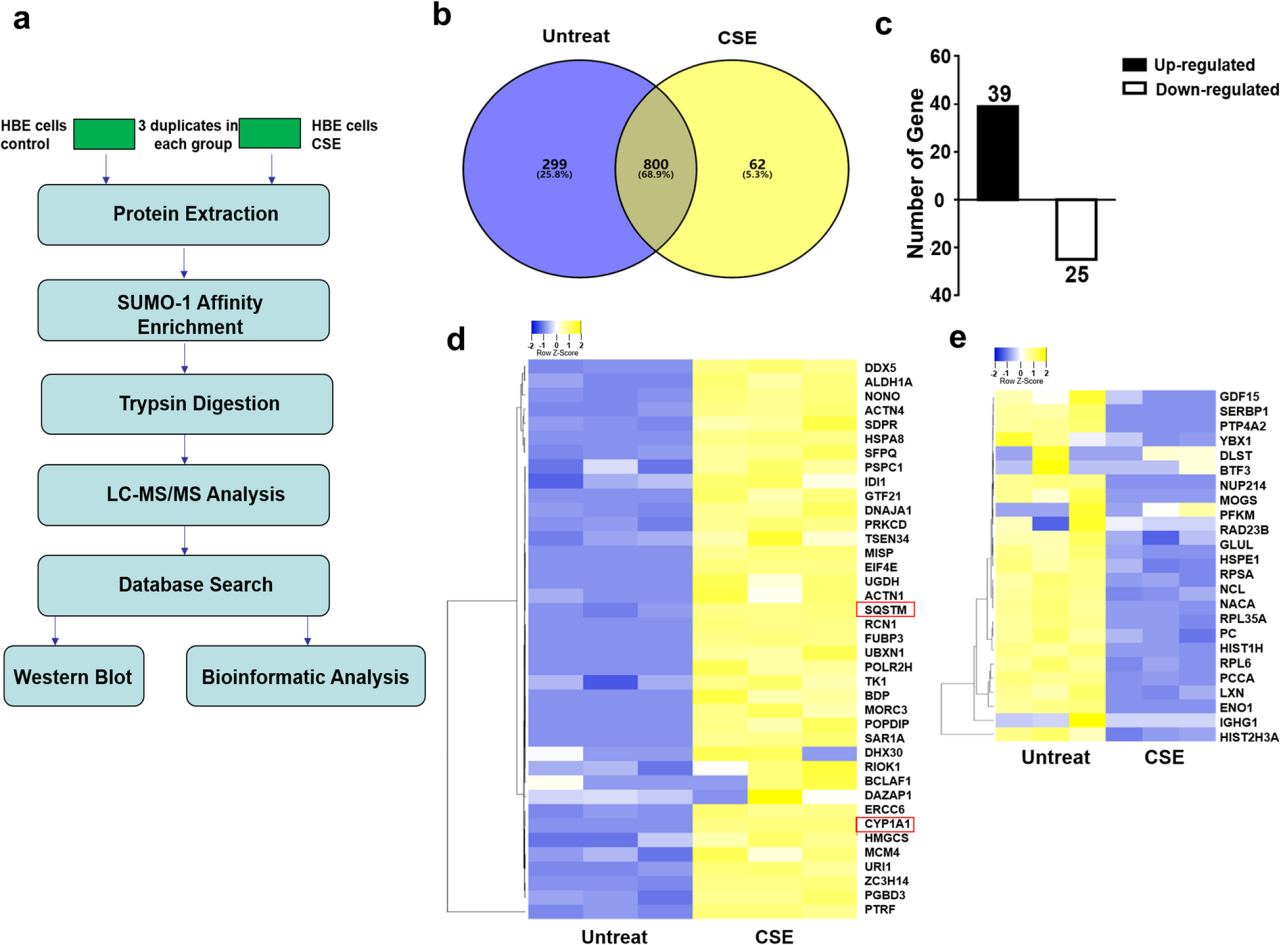
Strategy and results for comparative proteomics analysis of HBEs following CSE insult. **a** Experimental strategy and technical route for the quantitative proteomics analysis. In the LC-MS/MS analysis, two independent samples with or without CSE treatment were prepared. The proteins were digested with trypsin followed by co-immunoprecipitation using the SUMO-1 affinity columns. Each sample were undergone three parallel LC-MS/MS analyses to acquire quantitative proteomic data. **b** Venn diagram illustrating the overlap of identified proteins between control and CSE challenged HBEs. **c** A bar graphic figure showing 39 up-regulated proteins (2-fold changes) and 25 down-regulated proteins (2-fold changes). **d** A heatmap for the abundance of up-regulated (fold change ≥2) substrates. **e** A heatmap showing the abundance of the down-regulated (fold change ≤0.05) substrates

Next, we sought to conduct comparative proteomic analysis of SUMOylated substrates between CSE insulted and control HBEs. For this purpose, HBEs were treated with 20% CSE or control vehicle as described earlier. The harvested cells were then employed for immunoprecipitation of SUMOylated proteins using the SUMO-1 affinity columns, and the resulting products (two independent samples for each group) were subjected to LC-MS/MS analysis, and each sample were carried out with three replications, followed by database searching to identify protein identities (Fig. 2a). We assume that CSE stimulates HBEs to undergo a SUMOylation turnover (changes of SUMOylation patterns of its substrates and SUMOylation levels for a particular substrate), which may provide critical information to the understanding of COPD pathogenesis.

A total of 1201 SUMO-1 substrate proteins were identified from the above analysis **(**Table 1**)**. To acquire high-throughput quantitative results, the proteins with consistent fold changes in at least two of the three replications were included for the analysis, by which quantitative results were obtained from 847 SUMO-1 substrate proteins (Table 1). Among which, 299 were unique substrates for the untreated control cells, and 62 substrates were specific for the CSE challenged cells, while the rest of 800 proteins were shared by both CSE treated and untreated HBEs **(**Fig. 2b**)**. A cut off was then set up, and in which, a quantitative ratio over 2.0 was considered as upregulated substrates, while a quantitative ratio below 0.5 was regarded as downregulated substrates. This cut off allowed us to identify 39 upregulated proteins and 25 downregulated proteins in CSE treated HBEs **(**Fig. 2c**,** Table 2 and Tables 3 and 4**)**. A heatmap was finally generated to present the up- or downregulated substrates in CSE challenged HBEs as compared to the control cells **(**Fig. 2d and e**)**. In conclusion, the LC-MS/MS data demonstrated that CSE treatment induced significant changes in terms of SUMOylated substrate profiles, which were consistent with our Western blot results **(**Fig. 1d**)**.

**Table 1:** MS/MS identified summary

Total spectrums	Matched Spetrums	Peptides	Identified Proteins	Quantifiable Proteins
164,959	36,497	7106	1201	847

**Table 2:** Differentially modified SUMO-protein summary

Comparison ID	Regulated type	1.5-fold change	2-fold change
CSE/untreat	up-regulated	93	39
	down-regulated	74	25

**Table 3:** List of up-regulated proteins in CSE-treated HBEs

Protein accession	Protein name	CSE/untreat Ratio	Regulated Type	Gene name
P04798	Cytochrome P450 1A1	Inf	Up	CYP1A1
P52434	DNA-directed RNA polymerases I, II, and III subunit RPABC3	Inf	Up	POLR2H
P06730	Eukaryotic translation initiation factor 4E	Inf	Up	EIF4E
Q96I24	Far upstream element-binding protein 3	Inf	Up	FUBP3
Q9NR31	GTP-binding protein SAR1a	Inf	Up	SAR1A
Q8IVT2	Mitotic interactor and substrate of PLK1	Inf	Up	MISP
Q14149	MORC family CW-type zinc finger protein 3	Inf	Up	MORC3
Q9BY77	Polymerase delta-interacting protein 3	Inf	Up	POLDIP3
Q15293	Reticulocalbin-1	Inf	Up	RCN1
A6H8Y1	Transcription factor TFIIIB component B″ homolog	Inf	Up	BDP1
Q04323	UBX domain-containing protein 1	Inf	Up	UBXN1
O60701	UDP-glucose 6-dehydrogenase	Inf	Up	UGDH
Q15233	Non-POU domain-containing octamer-binding protein	6.407	Up	NONO
Q8N328	PiggyBac transposable element-derived protein 3	6.302	Up	PGBD3
Q03468	DNA excision repair protein ERCC-6	5.058	Up	ERCC6
Q6PJT7	Zinc finger CCCH domain-containing protein 14	4.122	Up	ZC3H14
Q969G5	Protein kinase C delta-binding protein	3.748	Up	PRKCDBP
Q13501	Sequestosome-1	3.651	Up	SQSTM1
Q96EP5	DAZ-associated protein 1	3.331	Up	DAZAP1
P12814	Alpha-actinin-1	3.158	Up	ACTN1
Q13907	Isopentenyl-diphosphate Delta-isomerase 1	2.932	Up	IDI1
Q01581	Hydroxymethylglutaryl-CoA synthase, cytoplasmic	2.913	Up	HMGCS1
P23246	Splicing factor, proline- and glutamine-rich	2.843	Up	SFPQ
P04183	Thymidine kinase, cytosolic	2.84	Up	TK1
Q7L2E3	Putative ATP-dependent RNA helicase DHX30	2.773	Up	DHX30
P31689	DnaJ homolog subfamily A member 1	2.667	Up	DNAJA1
P11142	Heat shock cognate 71 kDa protein	2.584	Up	HSPA8
Q6NZI2	Polymerase I and transcript release factor	2.504	Up	PTRF
Q9BSV6	tRNA-splicing endonuclease subunit Sen34	2.433	Up	TSEN34
Q8WXF1	Paraspeckle component 1	2.356	Up	PSPC1
O95810	Serum deprivation-response protein	2.315	Up	SDPR
P47895	Aldehyde dehydrogenase family 1 member A3	2.304	Up	ALDH1A3
O94763	Unconventional prefoldin RPB5 interactor 1	2.302	Up	URI1
P17844	Probable ATP-dependent RNA helicase DDX5	2.222	Up	DDX5
Q9BRS2	Serine/threonine-protein kinase RIO1	2.191	Up	RIOK1
Q9NYF8	Bcl-2-associated transcription factor 1	2.13	Up	BCLAF1
P78347	General transcription factor II-I	2.059	Up	GTF2I
P33991	DNA replication licensing factor MCM4	2.055	Up	MCM4
O43707	Alpha-actinin-4	2.043	Up	ACTN4

**Table 4:** List of down-regulated proteins in CSE-treated HBEs

Protein accession	Protein name	CSE/untreat Ratio	Regulated Type	Gene name
Q13724	Mannosyl-oligosaccharide glucosidase	0	Down	MOGS
P35658	Nuclear pore complex protein Nup214	0	Down	NUP214
Q8NC51	Plasminogen activator inhibitor 1 RNA-binding protein	0	Down	SERBP1
Q12974	Protein tyrosine phosphatase type IVA 2	0	Down	PTP4A2
P01857	Ig gamma-1 chain C region	0.052	Down	IGHG1
P61604	10 kDa heat shock protein, mitochondrial	0.117	Down	HSPE1
P19338	Nucleolin	0.17	Down	NCL
P06733	Alpha-enolase	0.27	Down	ENO1
Q99988	Growth/differentiation factor 15	0.283	Down	GDF15
Q13765	Nascent polypeptide-associated complex subunit alpha	0.288	Down	NACA
P15104	Glutamine synthetase	0.336	Down	GLUL
P20290	Transcription factor BTF3	0.337	Down	BTF3
P05165	Propionyl-CoA carboxylase alpha chain, mitochondrial	0.345	Down	PCCA
P08865	40S ribosomal protein SA	0.407	Down	RPSA
P16401	Histone H1.5	0.407	Down	HIST1H1B
P67809	Nuclease-sensitive element-binding protein 1	0.426	Down	YBX1
P54727	UV excision repair protein RAD23 homolog B	0.438	Down	RAD23B
Q13162	Peroxiredoxin-4	0.446	Down	PRDX4
Q02878	60S ribosomal protein L6	0.455	Down	RPL6
P36957	Dihydrolipoyllysine-residue succinyltransferase component of 2-oxoglutarate dehydrogenase complex, mitochondrial	0.463	Down	DLST
Q9BS40	Latexin	0.476	Down	LXN
P11498	Pyruvate carboxylase, mitochondrial	0.483	Down	PC
Q71DI3	Histone H3.2	0.487	Down	HIST2H3A
P08237	6-phosphofructokinase, muscle type	0.489	Down	PFKM
P18077	60S ribosomal protein L35a	0.49	Down	RPL35A

### Results for gene ontology analysis of SUMO1 substrates

**Fig. 3:**
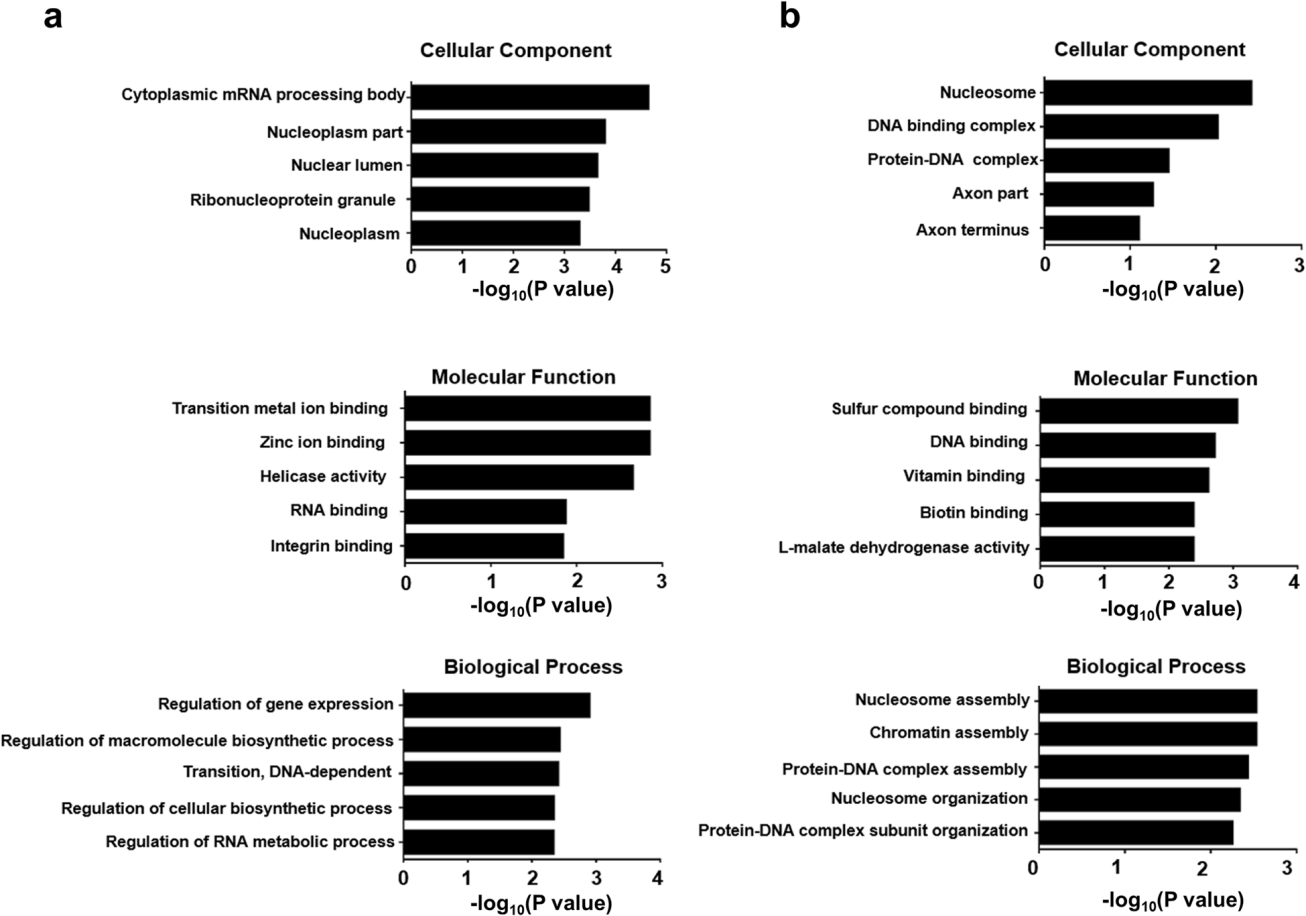
GO-based enrichment analysis of upregulated and downregulated proteins. In addition to cellular localization, the GO terms include Biological Process (BP), Cellular Component (CC) and Molecular Function (MF). **a** Analysis of upregulated proteins in BP, CC, and MF. **b** Analysis of downregulated proteins in BP, CC, and MF

The above characterized substrates were further examined by GO analysis and categorized according to their cellular localization. Of note, the most abundant proteins were derived from the nucleus (37%), followed by the cytoplasm (36%), mitochondria (10%), and extracellular region (6%) **(**Table 5**)**. In addition to cellular localization, GO terms include Biological Process (BP), Cellular Component (CC) and Molecular Function (MF) for functional analysis. The top upregulated substrates through analysis of GO terms are implicated in cytoplasmic mRNA processing body, transition metal ion binding and regulation of gene expression, respectively **(**Fig. 3a**)**. While the top downregulated substrates were involved in the regulation of nucleosome, sulfur compound binding and nucleosome assembly, respectively **(**Fig. 3b**)**.

**Table 5:** The summary of subcellular location of regulated and identified sumo-1 modified proteins

Subcellular Location	Number of Protein	Ratio
nucleus	64	37
cytoplasm	60	36
mitochondria	16	10
extracellular space	10	6
cytoplasm_nucleus	6	4
plasm	5	3
cytoskeleton	2	1
peroxisome	2	1
cytoplasm_mitochondria	1	1
endoplasmic reticulum	1	1

### Results for KEGG pathway analysis of substrates

**Fig. 4:**
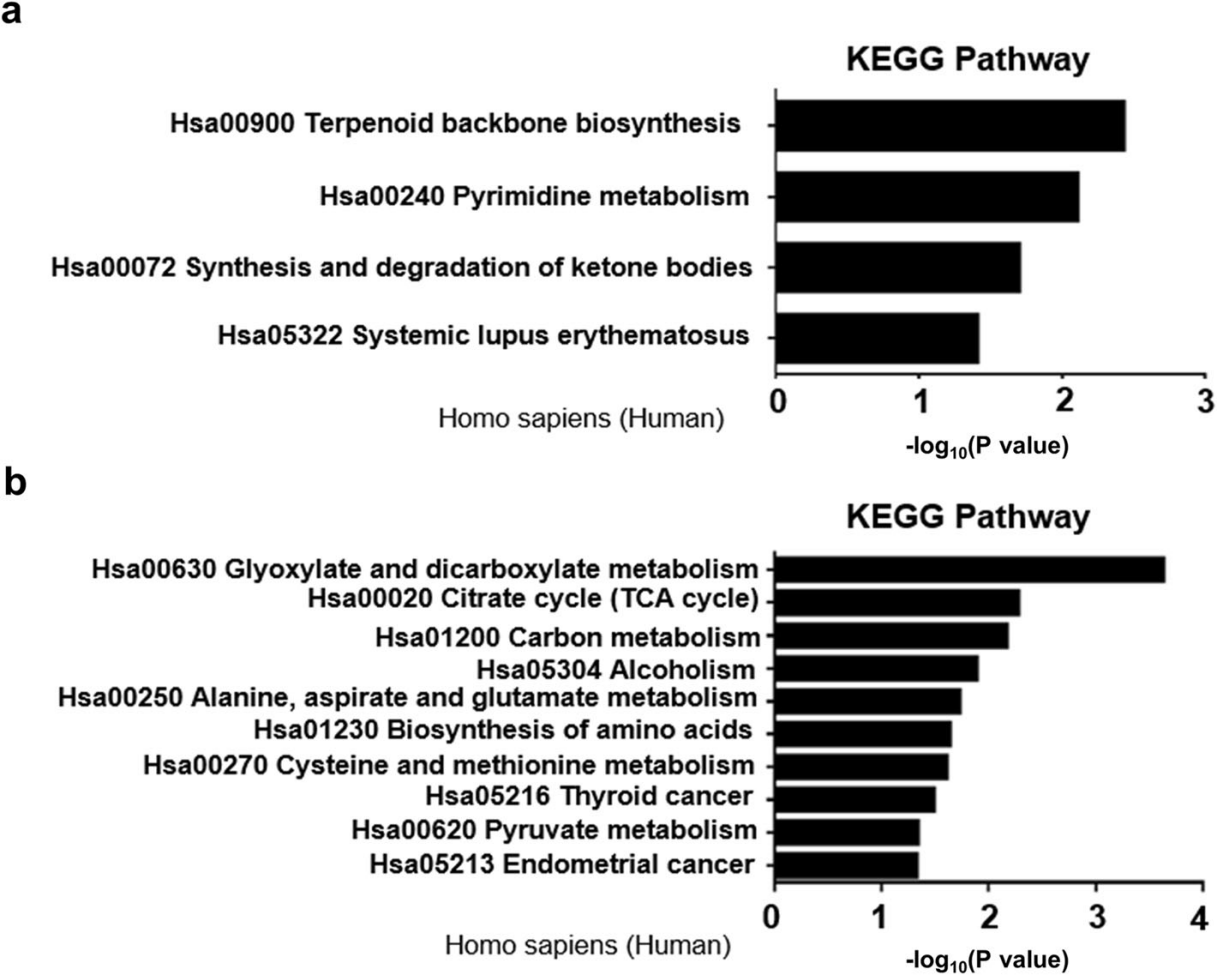
Results for the KEGG pathway-based enrichment analysis. **a** Summary of upregulated biochemical metabolic pathways. **b** Summary of downregulated biochemical metabolic pathways

KEGG significant enrichment was then conducted to identify the biochemical metabolic pathways of differentially SUMOylated substrates. KEGG analysis revealed 14 significant pathways with *P < 0.05*, including up-regulated pyrimidine metabolism, synthesis and degradation of ketone bodies, systemic lupus erythematosus **(**Fig. 4a**)**, and downregulated citrate cycle (TCA cycle), carbon metabolism, and biosynthesis of amino acids **(**Fig. 4b**)**. Particularly, the pathway relevant to the synthesis and degradation of ketone bodies was upregulated following CSE treatment. Recent studies have shown that ketone metabolism participates in the process of oxidative stress [19–21]. Ketogenic metabolism reduces oxidative stress after spinal cord injury in rats by inhibiting histone deacetylase (HDAC) [22], and a ketogenic diet and ketone salt supplementation can acutely and chronically enhance the expression of endogenous antioxidants and reduce oxidative stress in multiple tissues. Therefore, it is likely that the upregulated synthesis and degradation of ketone bodies could be a compensated effect resulted from CSE-induced oxidative stress.

### CSE enhances CYP1A1 SUMOylation to promote oxidative stress

**Fig. 5:**
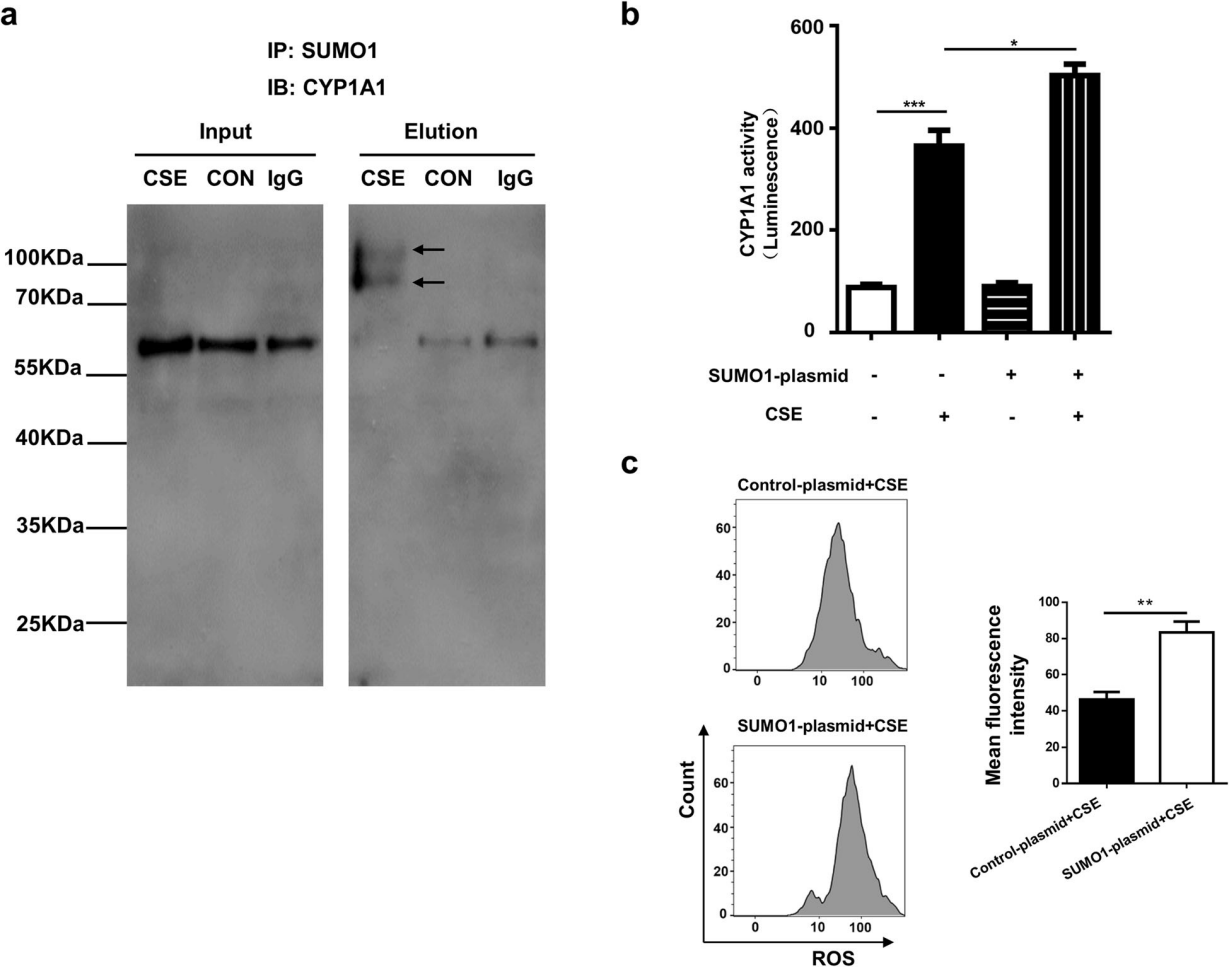
CSE induces CYP1A1 SUMOylation to regulate its enzymatic activity and ROS accumulation in HBEs. **a** Co-Immunoprecipitation results showing increased SUMOylation levels for CYP1A1 in CSE insulted HBEs. The input and elution samples were used for Western blot analysis. The SUMOylated bands of CYP1A1 are indicated by arrows. **b** Enzymatic activity analysis of CYP1A1 in HBEs with or without CSE treatment. **c** ROS analysis of HBEs transfected with control-plasmid or SUMO1-plasmid followed by CSE treatment. All experiments were conducted with 3 independent replications. The data are represented as the mean ± SEM. *, *P* < 0.05; **, *P* < 0.01 ***; *P* < 0.001

Given that a well-balanced SUMOylation pattern is indispensable for cell viability and against oxidative stress [23], we further analyzed the proteomic data with focus for the substrates relevant to antioxidant capability. Cytochrome P450 1A1 (CYP1A1), a member of the cytochrome P450 superfamily of enzymes that can use an electron from NADPH to reduce O_2_, leading to the production of H_2_O_2_ and a superoxide anion radical [24], was selected for further analysis. It was noted that its SUMOylation levels were significantly increased following CSE challenge **(**Fig. 2d**)**. To confirm this result, HBEs were challenged by CSE and then subjected to co-immunoprecipitation as described earlier, and the resulting precipitates were analyzed by Western blotting. Indeed, CYP1A1 is a substrate for SUMOylation, and CSE treatment significantly increased the levels of SUMOylated form of CYP1A1 **(**Fig. 5a, indicated by arrows). This result is consistent with published data that smoking induces CYP expression to increase oxidative stress [25].

Next, we intended to demonstrate the effect of SUMOylation on CYP1A1 enzymatic activity. To this end, 7-ethoxyresorufin-O-deethylase (EROD) assays were conducted to measure CYP1A1 enzymatic activity. It was noted that CSE remarkably increased the enzymatic activity of CYP1A1 **(**Fig. 5b**)**. More interestingly, once SUMO-1 was transfected into HBEs to enhance the SUMOylation of CYP1A1, CSE induced CYP1A1 activity was even higher **(**Fig. 5b**)** coupled with higher levels of ROS accumulation **(**Fig. 5c**)** and apoptosis (Supplementary Fig. 2). In addition, apoptosis can be rescued by treatment of antioxidant reagent Tempol (Supplementary Fig. 3). Taken together, our results demonstrate that CSE enhance CYP1A1 SUMOylation, thereby promoting oxidative stress and apoptosis in HBEs.

## Discussion

Cigarette smoking is the most common risk factor for COPD by inducing inflammation and damage in the airway epithelial cells. Therefore, understanding of the mechanisms underlying epithelial injury could be important to screen potential targets for developing therapeutic drugs against COPD in clinical settings. There is compelling evidence that posttranslational protein modifications function as a cellular regulatory mechanism in response to various stressful stimuli [26–28]. Among which, SUMOylation, one of the reversible posttranslational modifications, has recently been noted to play a critical role in the regulation of cellular processes including gene transcription, protein localization, DNA repair and cell cycle progression [29, 30]. Importantly, a well-balanced SUMOylation in substrates is essential for normal cellular behaviors, and altered SUMOylation predisposes to the risk for developing a large number of diseases such as cancer, neurodegenerative disorders (e.g., Alzheimer’s, Parkinson’s, and Huntington’s disease), diabetes and so on [31–36]. However, the exact impact of SUMOylation on the regulation of airway inflammation and epithelial damage in smoking subjects remains almost completely unknown.

In the present report, we conducted studies in HBEs to assess the relationship between CSE insult and the changes of SUMOylation profile. Interestingly, CSE specifically induced the expression of SUMO1 and Ubc9 in HBEs, but without a significant impact on the expression of SUMO2/3, SENP3 and SENP7. Next, we employed CSE to stimulate HBEs followed by immunoprecipitation of SUMOylated substrates along with LC-MS/MS analysis to obtain the accurate quantification data on the changes of SUMOylated proteins. We found that CSE induced HBEs to undergo a SUMOylation turnover as manifested by the changes of SUMOylated substrates and SUMOylation levels for a particular substrate. Specifically, we identified 39 up-regulated and 25 down-regulated proteins in CSE stimulated HBEs. These proteins were then annotated and enriched into different pathways through the Gene Ontology and KEGG analysis, respectively. Interestingly, our data revealed that those substrates with upregulated SUMOylation levels were involved in cytoplasmic mRNA processing body, transition metal ion binding and gene expression, while those substrates relevant to nucleosome and sulfur compound binding were down-regulated. The KEGG analysis of quantified proteins further indicated that the substrates with enhanced SUMOylation were enriched in ketone and pyrimidine metabolism, while those with decreased SUMOylation were implicated in TCA cycle, carbon metabolism and biosynthesis of amino acids. There is feasible evidence that ketone metabolism is involved in the process of oxidative stress induction [19, 21], and therefore, upregulation of SUMOylated substrates relevant to ketone metabolism following CSE challenge could be associated with ROS accumulation along with the induction of oxidative stress.

Indeed, some of the identified substrate proteins were recognized to be involved in the induction of oxidative stress, which may serve as critical risk factors contributing to the development of COPD [37, 38]. Particularly, CYP1A1, a member of the cytochrome P450 superfamily, was identified with enhanced SUMOylation in HBEs following CSE stimulation. Previous studies suggest that CYP1A1 could enhance oxidative stress by generating excessive ROS [39, 40]. We first confirmed that CYP1A1 can be SUMOylated, and SUMOylation enhances its catalytic activity as evidenced by the increased EROD signal along with higher ROS accumulation. Collectively, our data support that CSE induce oxidative stress not only by inducing the expression of CYP1A1, but also by enhancing its SUMOylation to increase its functionality.

Previous studies from other groups and ours have demonstrated convincing evidence that SUMOylation provides protection for cells against oxidative stress [11, 41, 42]. For example, SUMOylation of Nrf2 promotes its transcriptional activity, thereby protecting pancreatic beta cells from oxidative stress through enhanced capacity for detoxification of ROS [14]. In sharp contrast, our current data support that enhanced SUMOylation of CYP1A1 promotes its enzymatic activity along with increased ROS accumulation following CSE challenge in HBEs. This discrepancy is likely caused by the cell type differences and pathological stimuli, as each type of cells manifest different types of proteins for SUMOylation following stimulation. In support of this notion, our proteomic data revealed that NRF2 was SUMOylated in the pancreatic beta cells following inflammatory cytokine stimulation [14], while we failed to detect SUMOylated NRF2 in HBEs after CSE insult, rather CYP1A was found to be SUMOylated. In general, as aforementioned, a well-balanced SUMOylation pattern and levels of substrate proteins are essential for the cell viability and functionality. As a result, the overall impact of SUMOylation on the regulation of oxidative stress in a particular cell type may depend on the specific pathological conditions encountered (e.g., CSE insult in airway epithelial cells).

Of note, there are also some shortcomings in our present study. Although we have predicted by bioinformatics tools that Lys267 and 487 (K267 and K487) could be the SUMOylation sites, we failed to provide direct experimental evidence that these two lysine residues are the actual SUMO-conjugating sites, which would be our focus in the future studies. Additionally, P62, (also named Sequestosome1) also manifested with enhanced SUMOylation in HBEs following CSE insult. There is evidence that the upregulation and/or reduced degradation of P62 are implicated in tumor formation, cancer progression, and induction of oxidative stress as well as resistance to chemotherapy [43, 44]. However, the impact of SUMOylated P62 on CSE-induced airway epithelial injury is yet to be addressed.

## Conclusion

In summary, our investigation suggests that CSE induce oxidative stress in airway epithelial cells. CSE could promote the SUMOylation of CYP1A1, thereby enhancing ROS production to exacerbate oxidative stress. CSE also upregulates ketone metabolism to promote oxidative stress and to induce airway epithelial injury. Importantly, our study generated a protein SUMOylation profile, which could provide critical information for better understanding of the mechanisms underlying COPD.

## Supplementary information

**Additional file 1: Supplement Fig. 1.** Results for optimized CSE dose and exposure time. **(a)** Western blot results for SUMO1 expression following different percentage of CSE insult in HBEs. **(b)** Western blot results for SUMO1 expression following 20% CSE challenge with different time points. The data are represented as the mean ± SEM of 3 independent replications. *, *P* < 0.05; **, *P* < 0.01 ***; *P* < 0.001.

**Additional file 2: Supplement Fig. 2.** Apoptosis analysis of SUMO1 or control plasmid transfected HBEs following CSE insult. Annexin V^+^ 7-AAD^−^ represents early apoptotic HBEs, while Annexin V^−^ 7-AAD^+^ represents late stage apoptotic HBEs. The data are represented as the mean ± SEM (*n* = 3). *, *P* < 0.05; **, *P* < 0.01 ***; *P* < 0.001; ****, *P* < 0.0001.

**Additional file 3: Supplement Fig. 3**. CSE insult significantly induces HBEs to undergo apoptosis, which is attenuated by the antioxidant agent Tempol. Annexin V^+^ 7-AAD^−^ represents early apoptotic HBEs and Annexin V^−^ 7-AAD^+^ represents late stage apoptotic HBEs. The data are represented as the mean ± SEM (n = 3). *, *P* < 0.05; **, *P* < 0.01 ***; *P* < 0.001; ****, *P* < 0.0001.

## Data Availability

All data analyzed during this study are included in this article and its figures and tables. Additional data may be available from the corresponding author upon reasonable request.

## Abbreviations

COPD: Chronic obstructive pulmonary disease
CSE: Cigarette smoke extract
HBEs: Human bronchial epithelial cells
PTM: Posttranslational modification
SUMO: Small ubiquitin-like modifier
NRF2: Nuclear factor-erythroid 2-related factor 2
SENP3: SUMO-specific protease 3
SENP7: SUMO-specific protease 7
EROD: 7-ethoxyresorufin-O-deethylase
CYP1A1: Cytochrome P450 1A1
DMEM: Dulbecco’s Modified Eagle’s Medium
GO: Gene Ontology
KEGG: Kyoto Encyclopedia of Genes and Genomes
ROS: Reactive oxygen species
HDAC: Histone deacetylase
