# Supplementary figures and images for: Cigarette smoke extract stimulates bronchial epithelial cells to undergo a SUMOylation turnover
Source: BMC Pulm Med. 2020 Oct 23;20:276. doi: 10.1186/s12890-020-01300-w (PMC7584069; doi:10.1186/s12890-020-01300-w)

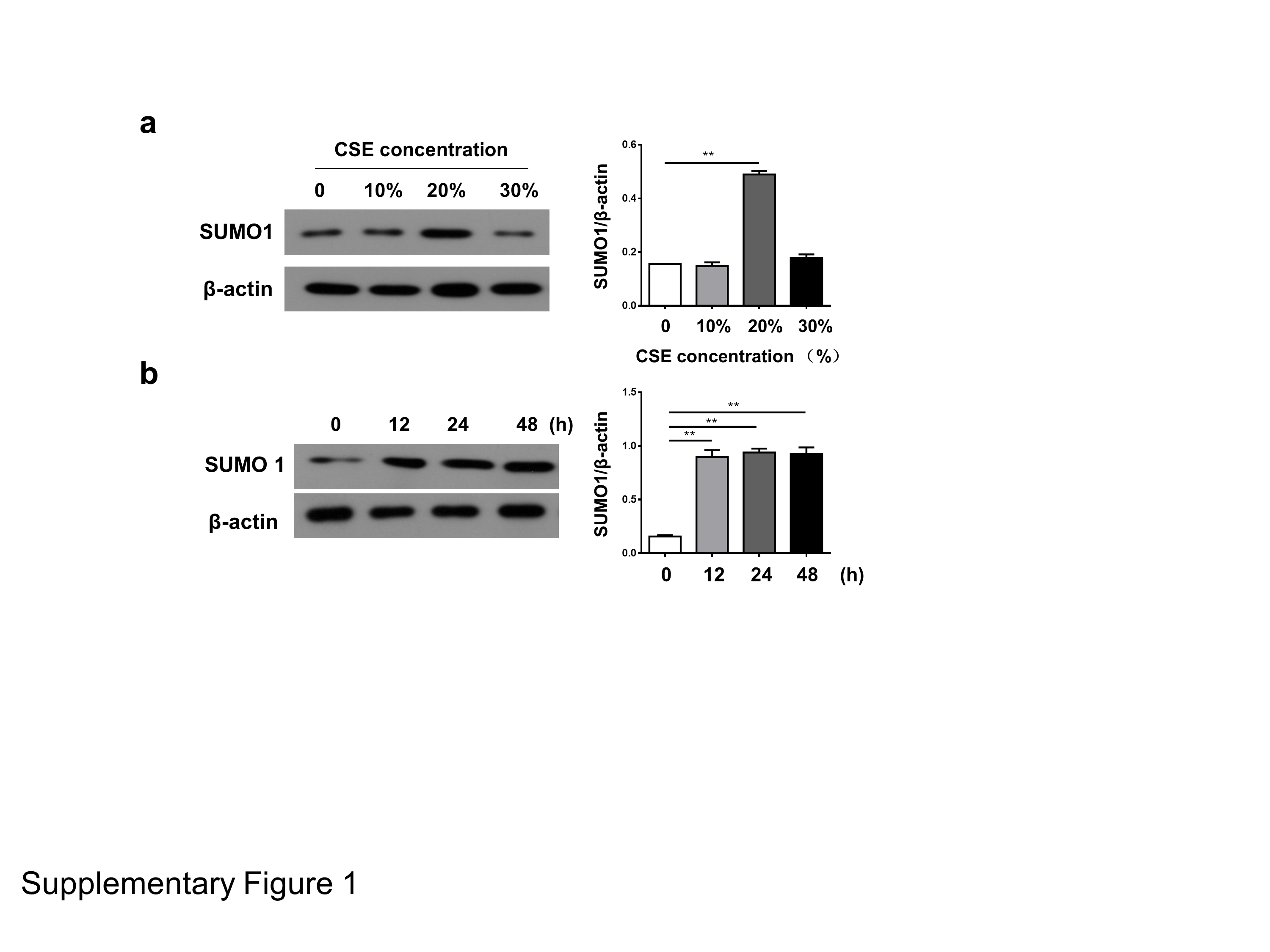

Supplement: Supplementary file 1 — Additional file 1: Supplement Fig. 1. Results for optimized CSE dose and exposure time. (a) Western blot results for SUMO1 expression following different percentage of CSE insult in HBEs. (b) Western blot results for SUMO1 expression following 20% CSE challenge with different time points. The data are represented as the mean ± SEM of 3 independent replications. *, P < 0.05; **, P < 0.01 ***; P < 0.001. [file 12890_2020_1300_MOESM1_ESM.tif]

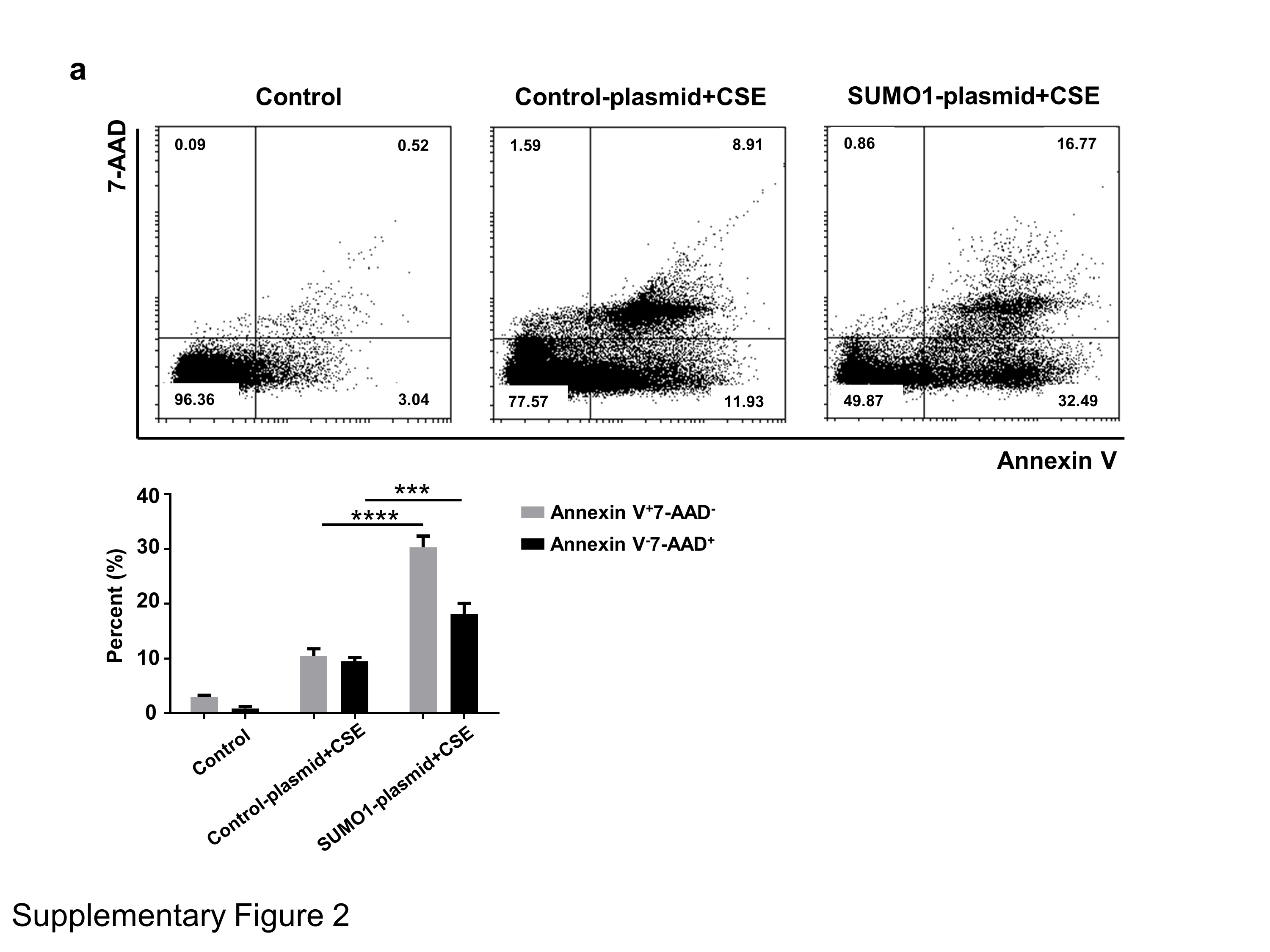

Supplement: Supplementary file 2 — Additional file 2: Supplement Fig. 2. Apoptosis analysis of SUMO1 or control plasmid transfected HBEs following CSE insult. Annexin V+ 7-AAD− represents early apoptotic HBEs, while Annexin V− 7-AAD+ represents late stage apoptotic HBEs. The data are represented as the mean ± SEM (n = 3). *, P < 0.05; **, P < 0.01 ***; P < 0.001; ****, P < 0.0001. [file 12890_2020_1300_MOESM2_ESM.tif]

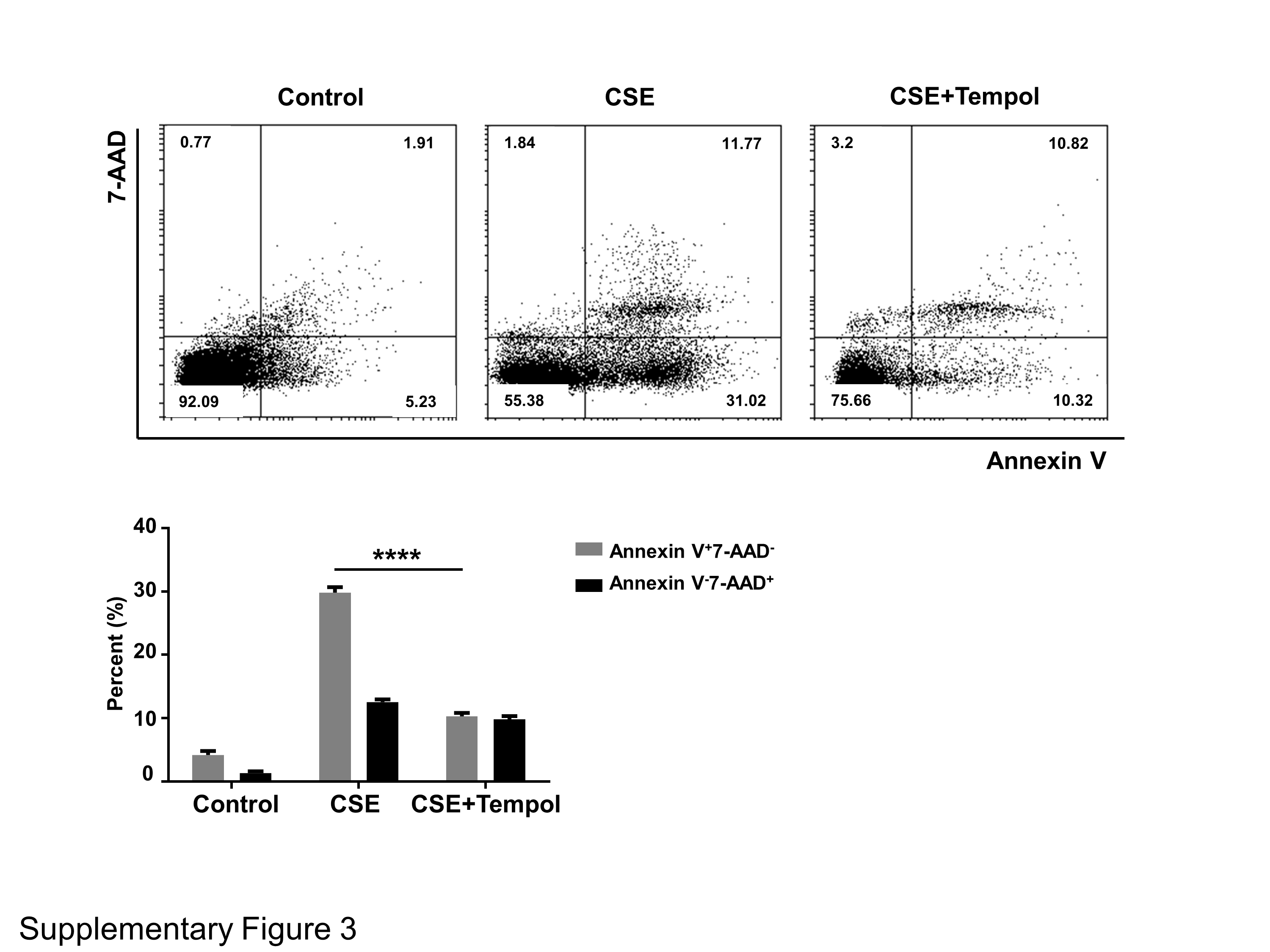

Supplement: Supplementary file 3 — Additional file 3: Supplement Fig. 3. CSE insult significantly induces HBEs to undergo apoptosis, which is attenuated by the antioxidant agent Tempol. Annexin V+ 7-AAD− represents early apoptotic HBEs and Annexin V− 7-AAD+ represents late stage apoptotic HBEs. The data are represented as the mean ± SEM (n = 3). *, P < 0.05; **, P < 0.01 ***; P < 0.001; ****, P < 0.0001. [file 12890_2020_1300_MOESM3_ESM.tif]
